# Bridging the Gender Gap in Cardiovascular Medicine: Addressing Drug Intolerances and Personalized Care for Women with Angina/Ischemia with Non-Obstructive Coronary Artery Disease

**DOI:** 10.3390/jcdd11120381

**Published:** 2024-11-28

**Authors:** Johanna McChord, Peter Ong

**Affiliations:** Robert Bosch Krankenhaus, Department of Cardiology and Angiology, Auerbachstr. 110, 70376 Stuttgart, Germany

**Keywords:** gender medicine, sex-based differences, non-obstructive coronary artery disease, ANOCA, INOCA, coronary microvascular dysfunction, drug intolerance, antianginal medication, personalized medicine

## Abstract

Gender medicine has increasingly underscored the necessity of addressing sex-based differences in disease prevalence and management, particularly within cardiovascular conditions and drug intolerance. Women often present cardiovascular diseases distinctively from men, with a higher prevalence of non-obstructive coronary artery disease and varied ischemic manifestations, such as coronary microvascular dysfunction and epicardial or microvascular coronary spasm. This disparity is further exacerbated by elevated drug intolerance rates among women, influenced by hormonal, genetic, and psychosocial factors. The 2024 ESC guidelines for managing chronic coronary syndromes stress the need for personalized approaches to treat angina and ischemia with non-obstructive coronary artery disease (ANOCA/INOCA), recommending a combination of antianginal medications. Despite standard treatments, up to 40% of ANOCA/INOCA patients experience refractory angina, necessitating a multifaceted approach that often involves multiple antianginal drugs, which can increase the likelihood of drug intolerances. Future research should focus on including women in drug studies and addressing sex-specific differences, while healthcare providers must be equipped to manage gender-specific drug intolerances. Enhanced awareness, individualized treatment strategies, and gender-sensitive healthcare policies are crucial for improving outcomes and bridging the gender gap in cardiovascular medicine.

## 1. Gender Differences in Cardiovascular Medicine

In the era of personalized medicine, gender medicine has emerged as a crucial aspect, addressing how differences between men and women impact disease prevalence and management. Gender medicine explores variations that traditional medicine often overlooks, but which are highly significant, such as differences in immune system function, cardiovascular responses, metabolism, and drug metabolism. Additionally, it considers how socio-cultural factors, including lifestyle, stress, and environmental influences, affect biological processes through the epigenome.

**Figure 1:**
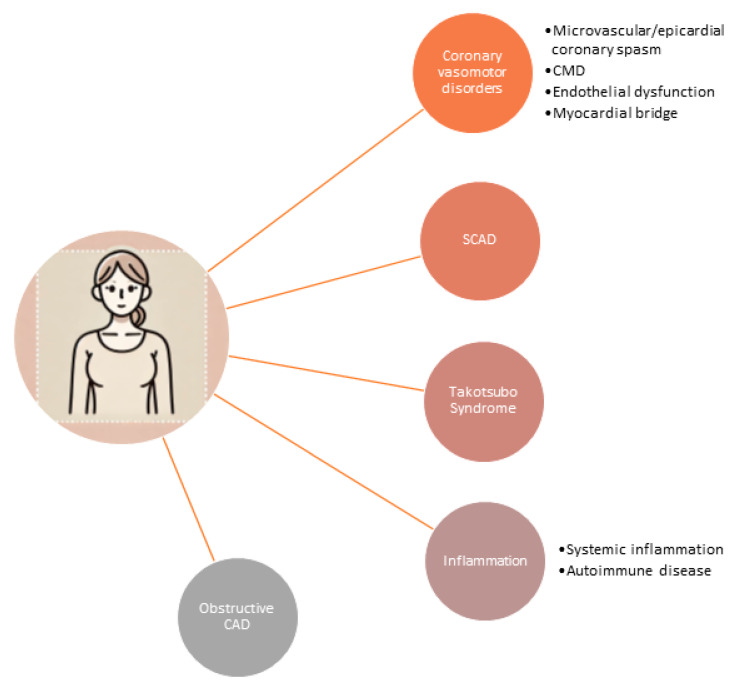
Spectrum of conditions in women with ischemic heart diseases. CMD: coronary microvascular dysfunction; SCAD: spontaneous coronary artery dissection; CAD: coronary artery disease.

These gender disparities are notably evident in various common diseases, particularly cardiovascular conditions [1]. Research into sex- and gender-specific cardiovascular disease (CVD) has significantly advanced our understanding of coronary artery disease (CAD) in women, revealing that it encompasses more than traditional atherosclerosis [2,3]. Women with ischemic heart disease (IHD) present a broader spectrum of conditions (Figure 1), including coronary vasomotor abnormalities, spontaneous coronary artery dissection (SCAD), Takotsubo syndrome, and inflammatory or autoimmune disorders, in addition to atherosclerotic obstructive CAD [4].

A key feature of IHD in women is the higher prevalence of angina, compared to men. Women also tend to have a lower burden of obstructive CAD, as observed through angiography, and face a worse prognosis overall [5]. Stress has been identified as a significant risk factor for myocardial infarctions in women, as shown in the VIRGO study [6], and diabetes further complicates this scenario [7]. Diabetes increases the risk of cardiovascular disease in women by 5–7 times with age, compared to 3–4 times in men, thus equalizing the absolute risk between genders. This increased risk is likely exacerbated by additional comorbidities such as obesity, chronic inflammation, and unfavorable changes in coagulation and endothelial function [7].

Premenopausal women generally experience lower rates of hypertension and lower lipid levels compared to men of the same age. However, this pattern shifts after menopause, with both hypertension and lipid levels rising significantly. Additionally, the overall burden of atherosclerosis increases as women age. While premenopausal women have a low risk of developing atherosclerosis, women between the ages of 45 and 65 face a moderate risk, with a greater likelihood of functional CAD, type II acute coronary syndromes (ACS), or outward remodeling of the coronary arteries, rather than obstructive CAD. As women age further, the risk of obstructive CAD increases, highlighting the protective cardiovascular role of estrogen, which diminishes after menopause [8].

Specialized women’s heart centers play a pivotal role in improving the diagnosis, treatment, and follow-up care for women with myocardial infarction with non-obstructive coronary arteries (MINOCA), helping to bridge significant knowledge gaps in this area [9]. A one-year prospective follow-up study [10] from a Canadian Women’s Heart Center (WHC) evaluated 154 women with non-obstructive CAD, 42 of whom were diagnosed with MINOCA. At baseline, the majority of these patients did not have a specific diagnosis. Through comprehensive investigations at the WHC, 60% of patients with MINOCA received a new or revised diagnosis, with coronary vasospasm being the leading diagnosis, identified in 60% of cases. After one year of care at the WHC, participants reported significant improvements in chest pain, quality of life, and mental health. These findings underscore the importance of specialized care centers in addressing the unique cardiovascular needs of women, particularly those with non-obstructive coronary artery conditions, by providing tailored diagnostic and therapeutic strategies that lead to better health outcomes. Current risk scores, based predominantly on male populations, do not accurately predict cardiovascular risk in women. This gap underscores the necessity for developing sex-specific biomarker ranges and risk-stratification tools to improve diagnosis, treatment, and follow-up for female patients [11].

Another important yet underexplored area in cardiovascular medicine is the interplay between cardiovascular diseases and comorbidities in women. One example is the link between angina in women without obstructive CAD and migraines. Siak et al. [12] found that migraines are prevalent among women with suspected ischemia and no obstructive CAD, and those with a migraine history more often report severe angina, compared to women without migraines. Notably, coronary vascular dysfunction identified through coronary function testing does not seem to correlate with the presence of migraines.

## 2. Multiple Drug Intolerance Syndrome

Adverse drug reactions (ADRs) are relatively common, making accurate identification of a patient’s allergy status crucial during medical history taking. The British Society for Allergy and Clinical Immunology defines drug allergies as ADRs associated with an immune mechanism, distinguishing them from pseudo-allergic, idiosyncratic, or intolerance reactions. Multiple Drug Intolerance Syndrome (MDIS) refers to patients who experience adverse reactions to three or more drugs without an immunological basis.

MDIS poses significant clinical challenges, as patients may develop severe reactions to multiple drugs, leading clinicians to avoid these medications and opt for less effective alternatives. The underlying mechanisms of MDIS are not well understood, but potential factors include nonspecific histamine release and psychological influences, such as the nocebo effect [13]. Patients with MDIS often present with elevated anxiety [14] and somatic symptoms [15], making it difficult to differentiate between genuine allergies and psychosomatic responses. Most recorded drug “allergies” are not IgE-mediated and may not be reproducible upon rechallenge [16]. Consequently, MDIS can restrict treatment options as patients become increasingly wary of new medications. Understanding MDIS better could provide insights into drug intolerance mechanisms and improve clinical management strategies.

A large-scale retrospective study in the UK revealed that 4.9% of patients with documented drug allergies had intolerances to three or more drugs [13]. This study uniquely compared MDIS patients to a control group presumed to have true IgE-mediated hypersensitivity, identifying specific risk factors for MDIS, rather than single-drug allergies. It found that women were significantly more affected by MDIS, being twice as likely as men to develop the condition. In addition to sex, MDIS was associated with older age, multiple comorbidities, and frequent hospital admissions. However, once comorbidities were accounted for, age itself was not an independent predictor, suggesting that comorbidities, which increase with age, play a more critical role in MDIS development. The study confirmed that comorbidities, particularly in older women, were strong predictors of MDIS. Factors such as weight, ethnicity, and socioeconomic deprivation had no significant impact.

A large-scale US study conducted between 2008 and 2015, and involving approximately 750,000 patients, found a 6.4% prevalence of MDIS [14]. Common drugs implicated included penicillins, opiates, sulfonamides, NSAIDs, cephalosporins, macrolide antibiotics, and radiocontrast agents. Angiotensin-converting enzyme inhibitors and statins were also frequently involved. This study highlighted a strong association between MDIS and psychiatric conditions such as anxiety and depression. The likelihood of anxiety increased with the number of drug intolerances, while depression was significantly associated with MDIS, increasing the odds by 50%. Psychiatry visits were notably more common among MDIS patients compared to those without drug allergies, a finding consistent across sensitivity analyses. Although selective serotonin reuptake inhibitors (SSRIs) are occasionally reported as causing intolerances, only a small percentage of MDIS patients reported issues with these medications. This underscores the need for specialized psychiatric support for MDIS patients, particularly when introducing new medications.

Table 1 provides a comprehensive overview of the typical adverse reactions associated with commonly prescribed cardiovascular drugs, detailing the variations observed between sexes.

## 3. Possible Causes of High Prevalence of MDIS in Women

The higher prevalence of MDIS in women can be attributed to a complex interplay of biological, hormonal, psychological, and behavioral factors.

One key factor is the influence of sex hormones, particularly estrogen, which can modulate immune responses and increase drug sensitivity in women [29]. Women generally exhibit stronger immune responses compared to men, including higher antibody production and B cell activity. This is partly due to the activity of immune-related genes on the X chromosome, some of which escape X-inactivation. These enhanced immune responses may heighten susceptibility to adverse drug reactions and autoimmune conditions.

Genetic variations in drug metabolism also play a key role in sex-specific pharmacokinetics, making women more susceptible to drug intolerances [30]. These differences stem from factors such as oral bioavailability, body fat composition and distribution, drug clearance, volume of distribution, absorption rates, plasma protein binding, urinary excretion, and metabolic pathways. A notable example is the pharmacokinetics of β-blockers like metoprolol and propranolol [31]. Due to differences in body composition, women typically have a smaller volume of distribution for these drugs, which can lead to slower clearance rates. Furthermore, β-blockers metabolized by the cytochrome P450 enzyme CYP2D6 are cleared faster in men, resulting in higher plasma concentrations in women and a greater likelihood of adverse effects [32].

Psychosocial factors also play a significant role. Women are more likely to experience anxiety and depression [33], conditions strongly associated with MDIS. Psychosomatic tendencies, including higher rates of somatization [34], may further contribute to greater sensitivity to medications and an increased frequency of reported adverse effects. Moreover, women’s higher healthcare utilization rates [35] result in exposure to a broader range of medications, increasing the likelihood of encountering adverse reactions. This increased exposure, coupled with a proactive approach to symptom reporting, may lead to a higher diagnosis rate of MDIS among women.

Additionally, the historical underrepresentation of women in clinical trials [29] has limited our understanding of sex-specific responses to medications. This disparity has contributed to a higher incidence of ADRs in women, highlighting the need for more inclusive sex-based analyses in clinical research.

**Figure 2:**
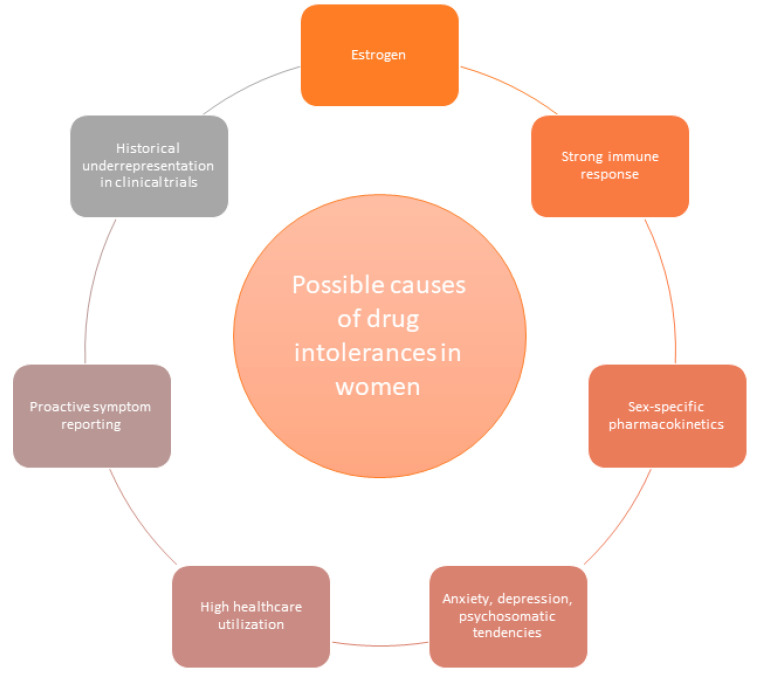
Possible causes of drug intolerances in women.

Overall, these biological, psychological, and social factors intertwine to explain the elevated prevalence of MDIS in women (Figure 2).

## 4. Drug Intolerances in ANOCA/INOCA Therapy

Drug intolerances present a significant challenge in managing ANOCA/INOCA (angina/ischemia with non-obstructive coronary artery disease), a condition observed in approximately 50% of patients undergoing diagnostic coronary angiography for suspected obstructive CAD. Unlike traditional CAD, in which plaque buildup obstructs coronary arteries, ANOCA/INOCA occurs despite the absence of major blockages. It is often attributed to functional coronary disorders, including epicardial or microvascular coronary spasms, or impaired microvascular vasodilation, known as coronary microvascular dysfunction (CMD). These issues restrict blood flow at the microvascular level, leading to symptoms similar to those seen in obstructive CAD.

Diagnosing IHD in women based on symptoms is challenging, as women often report non-cardiac symptoms. Taha et al. [36] analyzed data from 916 women in the WISE cohort (NCT 00000554) who underwent coronary angiography for suspected ischemia and completed a 65-item symptom questionnaire. Among them, 62% were identified as having suspected INOCA. Using logistic regression with a best-subsets approach, the researchers developed a 10-variable predictive model for INOCA. The model revealed that age ≤55 years, left-sided chest pain, chest discomfort, neck pain, and palpitations were positively associated with INOCA, while symptoms such as impending doom, and pain in the jaw, arms, or right hand were inversely associated. This model predicted INOCA, with ~72% accuracy, based on age and symptom profile. 

The 2024 ESC guidelines for the management of chronic coronary syndromes [37] underscore that symptoms of myocardial ischemia due to obstructive atherosclerotic CAD often overlap with those of coronary microvascular disease or vasospasm. They recommend, as a Class IB guideline, that persistently symptomatic patients with angina or angina equivalents despite medical treatment and with suspected ANOCA/INOCA and poor quality of life should undergo invasive coronary functional testing. This type of testing, which assesses coronary function at a detailed level, can identify potentially treatable causes of ANOCA/INOCA and provide a pathway to more tailored and effective care.

Through invasive coronary functional testing, approximately 90% of ANOCA/INOCA cases can be diagnosed, with microvascular and epicardial coronary spasm being the most prevalent underlying issues [38]. Notably, women represent around 88% of ANOCA/INOCA cases [39], reflecting a significant gender disparity in this condition. The treatment of anginal symptoms in ANOCA/INOCA patients is particularly complicated, due to the complex nature of the condition and limited evidence from large-scale randomized trials.

The CorMicA study [40] has demonstrated that a stratified antianginal therapy algorithm based on coronary functional testing can improve angina symptoms and quality of life compared to standard therapy. For patients with microvascular angina (MVA) and reduced coronary flow reserve (CFR) and/or increased index of microvascular resistance (IMR), treatment often includes β-blockers, calcium channel blockers (CCBs), ranolazine, and angiotensin-converting enzyme inhibitors (ACE-Is) [41]. Specifically, anti-ischemic therapy with amlodipine or ranolazine has been shown to significantly improve exercise capacity [42].

In patients with epicardial or microvascular coronary spasm, calcium antagonists are recommended as first-line therapy following acetylcholine (ACh) spasm provocation testing. In cases of severe vasospastic angina (VSA), higher dosages of calcium antagonists, such as 200 mg of diltiazem twice daily or even up to 960 mg daily, or a combination of non-dihydropyridine (e.g., diltiazem) and dihydropyridine CCBs (e.g., amlodipine) may be necessary. However, a small Dutch study [43] reported that oral diltiazem or placebo up to 360 mg/day did not substantially improve symptoms or quality of life, though diltiazem did reduce the prevalence of epicardial spasm. Nicorandil, a vasodilator with nitrate-like action and potassium-channel activation, may be an effective alternative despite frequent side effects [44]. Ranolazine, an antianginal agent that improves myocyte relaxation and ventricular compliance by reducing sodium and calcium overload, can be combined with first-line therapies [45]. Table 2 gives an overview of recommended first-line antianginal medication in ANOCA/INOCA.

Despite those medical treatments, up to 40% of ANOCA/INOCA patients continue to suffer from refractory angina, necessitating the use of multiple antianginal medications or off-label prescriptions [46]. Drug intolerances are frequently reported among these refractory angina patients, particularly women. Our clinical experience indicates that the female ANOCA/INOCA patients who report frequent drug intolerances are often those with microvascular coronary spasms. Unfortunately, the coronary microvasculature responds less effectively to nitrates, compared to epicardial coronary arteries, making sublingual nitroglycerin less beneficial for patients with microvascular coronary spasm [47]. To address these challenges, we recommend the following practical considerations:

1. Initiate lower drug doses in women, gradually increasing the dosage over a longer period to improve drug tolerance [48].
2. Consider ranolazine for patients experiencing adverse reactions like bradycardia or hypotension, as it does not significantly lower heart rate or blood pressure [49].
3. Incorporate supplements, such as magnesium, especially for patients with coronary spasm, to promote vascular relaxation without significant side effects [50].
4. Explore alternative therapies to improve quality of life, such as repurposing drugs like endothelin receptor antagonists or sGC stimulators/activators [46].
5. Consider non-pharmacological treatments for refractory ANOCA/INOCA patients, including spinal cord stimulation [51] or coronary sinus reducer implantation [52].

Despite the challenges faced by women with refractory angina, there is cause for optimism due to the ongoing development of novel therapies aimed at addressing the underlying pathology. These include coronary sinus reducers, CD34+ stem cell therapy, and emerging pharmacologic agents such as sGC stimulators and endothelin-receptor antagonists [53]. 

## 5. Outlook: Addressing Drug Intolerances in Women and Closing the Gender Gap

To improve the understanding and management of drug intolerances in women, a multifaceted approach is essential, with the goal of closing the persistent gender gap in healthcare. First and foremost, clinical research must prioritize the inclusion of women in studies of drug efficacy and adverse effects, moving beyond historical biases that have often excluded females from such trials. Tailoring clinical trials to account for sex-specific differences in pharmacokinetics and pharmacodynamics (especially in phase I and II drug development studies) will be crucial in developing safer and more effective therapies for women. Furthermore, preclinical research must address sex as a biological variable, focusing on the roles of hormones, genetic factors, and immune responses in driving drug intolerances.

Healthcare providers also need to be trained to recognize the unique manifestations of drug intolerance in women and adjust treatments accordingly. Increased awareness and education as to gender-specific responses to medication, particularly in complex conditions like ANOCA/INOCA, will allow for more personalized treatment plans that can minimize adverse reactions while optimizing therapeutic outcomes.

On a broader level, healthcare policies must support equitable access to care and encourage gender-sensitive approaches to drug prescribing and monitoring. By closing the gender gap with medical research, clinical practice, and health policy, we can ensure that women receive more accurate diagnoses and safer, more effective treatments, ultimately improving their quality of care and health outcomes [54,55,56,57].

## 6. Conclusions

The intersection of gender and cardiovascular medicine underscores the urgent need to address the distinct ways in which cardiovascular diseases and drug intolerances manifest in women. Women present with a broader spectrum of ischemic heart conditions and higher rates of drug intolerances, influenced by biological, hormonal, and psychosocial factors. The 2024 ESC guidelines reflect a shift towards personalized treatment strategies for ANOCA/INOCA, emphasizing the necessity of tailored therapies and a combination of medications to manage symptoms effectively. However, significant challenges remain, including the underrepresentation of women in clinical trials and the need for more accurate, gender-specific risk assessments. Closing the gender gap in research and clinical practice is imperative for developing safer, more effective treatments for women. By advancing gender-sensitive research, enhancing clinical training, and implementing equitable healthcare policies, we can improve diagnosis, treatment, and overall health outcomes for women with cardiovascular conditions and drug intolerances.

## Figures and Tables

**Table 1:** Summary table of cardiovascular drug classes, common adverse drug reactions, and sex differences.

Drug Class	Common Adverse Drug Reactions	Sex Differences in Adverse Drug Reactions	References
Statins	Muscle pain, myopathy, liver enzyme elevation, fatigue, gastrointestinal symptoms	Women have a higher risk of statin-associated muscle symptoms and higher rates of discontinuation due to adverse effects	[17,18]
Dihydropyridine Calcium Channel Blockers (CCBs) (amlodipine, nifedipine)	Edema, constipation, headache, dizziness, flushing	Edema more common in women	[19,20]
β-Blockers	Fatigue, bradycardia, cold extremities, depression, sexual dysfunction	Greater reduction in blood pressure and heart rate in women treated with metoprolol and propranolol	[19]
ACE Inhibitors	Cough, angioedema, hypotension, dizziness, renal impairment	Dry cough is 2-3 times more frequent in women; no sex difference for angioedema	[19,20,21,22]
Angiotensin II Receptor Blockers (ARBs)	Dizziness, hyperkalemia, hypotension, headache	Limited data on sex-specific intolerance rates; one analysis suggests no substantial gender difference	[23]
Diuretics	Electrolyte imbalances, dehydration, dizziness, gout	Greater risk of hypo-osmolarity, hypokalemia, hyponatremia, and arrhythmias in women, especially with thiazides	[19,24]
Antiplatelet Agents (e.g., aspirin, clopidogrel)	Gastrointestinal bleeding, dyspepsia	Increased risk of GI bleeding in older women (>70 yo) with aspirin; no evidence on sex differences with clopidogrel	[25,26]
Anticoagulants (e.g., warfarin, DOACs)	Bleeding, bruising, anemia	Increased bleeding risk in women; women need less warfarin per week than men	[19]
Class III antiarrhythmic drug (amiodarone)	thyroid dysfunction, photosensitivity, visual disturbance, bradyarrhythmia, sinus arrest, and hepatotoxicity	increased risk of bradyarrhythmia requiring pacemaker insertion and phototoxicity in women	[27,28]

**Table 2:** Overview of recommended first-line antianginal medication in ANOCA/INOCA.

Condition	First-Line Treatment	Additional Notes
MVA with Reduced CFR and/or Increased IMR	β-blockers, CCBs, ranolazine, ACE-Is	Ranolazine improves exercise capacity [42]
Epicardial or Microvascular Coronary Spasm	CCBs (may be combined with long-acting nitrates and/or ranolazine)	Study showed diltiazem reduced epicardial spasm but did not substantially improve symptoms or quality of life [43]
Severe Vasospastic Angina (VSA)	Higher dosages of CCBs (e.g., diltiazem up to 960 mg daily) or combination of non-dihydropyridine and dihydropyridine CCBs	Sublingual nitroglycerin spray may alleviate acute angina pectoris episodes
